# Correspondence between Simulator and On-Road Drive Performance: Implications for Assessment of Driving Safety

**DOI:** 10.3390/geriatrics1010008

**Published:** 2016-03-10

**Authors:** Nazan Aksan, Sarah D. Hacker, Lauren Sager, Jeffrey Dawson, Steven Anderson, Matthew Rizzo

**Affiliations:** 1 Department of Neurology, University of Iowa, Iowa City, IA 52242, USA; sarah-d-hacker@uiowa.edu (S.D.H.); steven-anderson@uiowa.edu (S.A.); 2 Department of Biostatistics, University of Iowa, Iowa City, IA 52242, USA; lauren-sager@uiowa.edu (L.S.); Jeffrey-dawson@uiowa.edu (J.D.); 3 Department of Neurological Sciences, University of Nebraska Medical Center, Omaha, NE 68198, USA; matthew.rizzo@unmc.edu

**Keywords:** older driver, relative validity, driving safety

## Abstract

Forty-two younger (Mean age = 35) and 37 older drivers (Mean age = 77) completed four similar simulated drives. In addition, 32 younger and 30 older drivers completed a standard on-road drive in an instrumented vehicle. Performance in the simulated drives was evaluated using both electronic drive data and video-review of errors. Safety errors during the on-road drive were evaluated by a certified driving instructor blind to simulator performance, using state Department of Transportation criteria. We examined the degree of convergence in performance across the two platforms on various driving tasks including lane change, lane keeping, speed control, stopping, turns, and overall performance. Differences based on age group indicated a pattern of strong relative validity for simulator measures. However, relative rank-order in specific metrics of performance suggested a pattern of moderate relative validity. The findings have implications for the use of simulators in assessments of driving safety as well as its use in training and/or rehabilitation settings.

## 1. Introduction

Older drivers are at increased risk for crashes and numerous studies have linked age-related declines in visual, motor, and cognitive function to decreased driver safety [1,2,3]. The “gold-standard” in evaluations of driving safety remain on-road drive tests which are expensive. These evaluations can also be unsafe for the driver and the examiner in the absence of well-established screening criteria that will accurately eliminate those who would not be safe behind the wheel based on functional declines. Driving simulation holds a particular promise in this regard. Simulation is a safe alternative to actual on-road tests in evaluating safety and road worthiness of at-risk populations before subjecting anyone to risks of a road test. In addition, it has the potential to be used in rehabilitation/training of at-risk drivers. The consensus on whether driving simulation has sufficient validity to inform real-world safety varies both by the perspective or goals adopted [4] and the driving simulator used (e.g., STISIM *versus* DriveSafety) [5,6].

The term *absolute validity* refers to a pattern of findings in which simulator performance closely aligns with performance in an on-road test in terms of numerical scores. Available evidence suggests that simulators have limited absolute validity [4]. Establishing absolute validity is difficult for a variety of different reasons and this form of validity may have limited utility if the goal is to evaluate the road worthiness of a driver. For example, absolute validity often requires simulating the identical layout and sequence of tasks to an on-road test. If the on-road test is carried out in in real-world traffic rather than a closed course, the possibility of establishing close alignment in numerical results across the two platforms is significantly diminished.

In contrast, the term *relative validity* refers to two types of evidence [4]. First, performance rank-ordering in the simulator and the on-road test should be significantly correlated [4,7]. Second, findings in each platform should produce similar inferences as a function of study design factors. An example of the first type of evidence would be total number of safety errors or demerit points in one platform shows significant positive correlations with comparable measures in the other platform [7]. An example of the second type of evidence would be to show similar inferences for any between-subject characteristics such as age group [4] and within-subject differences such as reduced driving safety while distracted compared to baseline driving [8]. In this second case, relative validity can be understood as replication of findings across the two platforms for similar performance metrics. For example, if on-road tests indicate that older drivers perform significantly worse than younger drivers in lane observance/keeping tasks, the simulator-based assessments should also point to significant mean differences in lane keeping for the age groups.

As opposed to absolute validity, findings suggest there is greater relative validity to driving simulation [4,9]. However, a closer examination of these findings often indicate that relative validity, when supported, is often examined for a specific subset of measures such as speed control [10,11] or lane position [11] and sometimes for overall safety metrics such as demerit points [12,13,14] or crash/citation history [15,16]. While overall safety metrics including crashes and total demerit points are valuable because of their inherent ecological and face validity, they have limited utility in gauging deficits or competencies for specific driving tasks such as changing lanes, stopping/braking, and turning among others. In fact, relative validity for specific driving tasks is essential to demonstrate if simulators hold the promise of being useful screening or training/rehabilitative tools [9].

**Figure 1:**
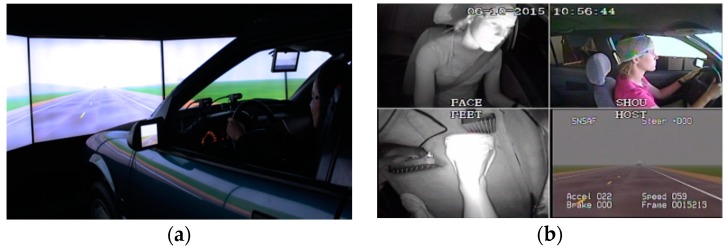
DriveSafety simulator: (**a**) view of the visual scene and driver from the outside; (**b**) view of the driver from inside the cabin.

**Figure 2:**
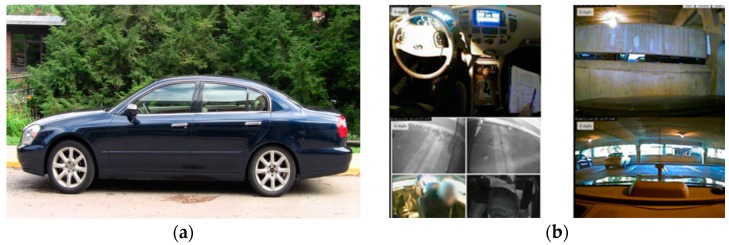
Instrumented vehicle: (**a**) outside view of the vehicle; (**b**) location of cameras from inside the cabin used by driving instructor to evaluate performance.

Our goal in this study was to inform relative validity of driving performance in specific driving tasks including lane observation, lane changing, stopping, turning, and controlling speed as well as overall safety among younger and older drivers. Majority of previous studies supporting relative validity have been conducted with STISIM [4,13,14]. In this study, we examined relative validity using a fixed base, full cabin 180 degree forward field of view DriveSafety simulator (DS600). We relied on both simulator vehicle data and judgment of coders regarding performance in each scenario from the videos. The participants also completed a 45-min on-road drive in an instrumented vehicle in real-traffic and had their safety errors evaluated by a certified driving instructor who was blind to performance in the simulator. Photographs of both the simulator and the instrumented vehicle are shown in Figure 1 and Figure 2 respectively. 

To address relative validity we examined the two types of evidence described earlier. First, we examined whether older drivers performed significantly worse than younger drivers in each driving task and overall performance scores in both the simulator and the on-road drive. We evaluated these age differences for patterns of replication across the two platforms. Weak evidence of relative validity would be obtained if significant age differences did not replicate across the two platforms for specific and overall performance metrics. An example of lack of replication would be that significant age difference in lane observance is found in the simulator but not the on-road drive. Conversely, strong evidence of relative validity would be obtained if age group differences replicated in each platform such that significant differences in one platform were significant in the other, and non-significant differences in one platform were also not significant in the other platform. In other words, evidence for strong relative validity requires that both platforms distinguish at-risk group of drivers from those not-at-risk similarly on specific driving tasks as well as overall safety metrics.

Second, we examined similarity in relative rank-ordering of performance both in terms of specific driving tasks and overall safety in the two platforms with correlations. Weak evidence of relative validity would be obtained if relative rank-order in performance for specific driving tasks were not significantly correlated across the two platforms or only overall performance metrics showed significant correlations. Conversely, strong evidence of relative validity would be obtained if both overall and specific performance metrics showed convergence across the two platforms. Finally, we also evaluated whether specific and/or overall safety metrics demonstrated strong relative validity in both set of analyses, one focused on discriminating performance as a function of risk-group and the other focused on alignment in general rank-order.

## 2. Methods

### 2.1. Sample

The sample included 42 younger and 37 older drivers. The younger drivers ranged from 25 to 50 years old (mean = 35 years), and consisted of 19 males and 23 females. The older drivers ranged from 66 to 87 years old (mean = 77 years), and consisted of 23 males and 15 females. Inclusion criteria included: (a) valid driver’s license; (b) minimum 10 years of driving experience; (c) driving at least 1 hour or 50 miles per week; (d) negative screen for dementia (Montreal Cognitive Assessment, MoCA > 18) [17]; and (e) corrected visual acuity better than 20/50. Twenty-four percent of the younger and 42% of the older drivers scored in the Mild Cognitive Impairment range, consistent with a broad sampling of cognitive function. Eighty-six percent of the sample was Caucasian. Educational achievement was distributed as follows: 7% had high school or less, 45% had less than college degree, 45% had a college degree or more, 3% did not indicate education level.

### 2.2. Procedure and Design

Following basic visual function and dementia screening, participants took a simulator drive (fixed base, full cabin with 180° FOV, DriveSafety RS600) to assess motion sickness. Of those tested in the simulator, 28 experienced symptoms of motion sickness and were not part of the 79 whose data were analyzed here. Those who experienced motion sickness did not differ in age, MoCA, or years of driving experience (min *p* < 0.090). Older drivers were not more likely to experience motion sickness compared to younger drivers, chi-square(1) = 0.566, *p* = 0.444. The remainder participated in detailed assessments of their cognitive function including processing speed, memory, and visuospatial construction during a second visit, not reported in this study. In four additional visits approximately scheduled 2 weeks apart, participants completed four simulated drives. The drives differed with respect to layout but were similar in terms of tile composition with respect to road culture, total time (15–18 min each), and number of hazards encountered. In another visit, participants drove an instrumented vehicle on the road through an 18-mile route that takes about 45 min to complete. A trained research assistant gave navigation instructions typical of on-road drives such as “make a right turn at the lights”. The route included a mixture of residential, suburban, rural, and highway roads, and included navigation tasks discussed in prior publications [2,18]. The videos of the drive were evaluated by a certified driving instructor for safety errors according Iowa Department of Transportation (IA-DOT) standards [18,19]. A total of 62 participants completed the on-road drive test. Reasons for missing data in the on-road drive included equipment malfunction or scheduling difficulties. Distribution of missing *versus* non-missing data in the on-road drive were similar for the two age groups, chi-square(1) = 0.343, *p* = 0.558.

### 2.3. Measures

Table 1 summarizes the measures extracted for the following driving tasks: lane change, lane keeping, speed control, stopping, turning, performance during incursions, and traffic sign compliance. The measures from the simulator included both and evaluations of video for safety in each scenario and vehicle data. Each video was reviewed twice by two different coders and any disagreements were resolved with discussion. Many of the measures listed in Table 1 from evaluation of videos for safety in simulator scenarios were binary judgments or counts, and because not every participant completed all four drives, these measures were transformed to proportions, e.g., proportion of rolling stops, or rates for count measures, e.g., rate of gap rejection during lane changes in each completed drive, and averaged across all available drives. Vehicle data from the simulator included additional measures extracted from drive files such as gap size, standard deviation of lane position. Finally, the specific measures listed for each driving task were z-transformed and averaged to form composite measures of performance in the simulator. High scores represented poor performance in each driving task. The measures obtained from the on-road drive were based on safety error classification scheme of the IA-DOT. The classification scheme for these errors has been described in prior publications extensively [2,18,19]. In the current study, we selected those errors that conceptually aligned with simulator measures and errors that prior research has shown to occur frequently including lane observance, lane change, speed control, turns, stopping, and traffic sign errors [18,19]. The last column of Table 1 highlights the types of safety errors noted in the classification scheme of IA-DOT that map unto the driving tasks. We did not specifically examine several additional error categories or driving tasks such as start-pulling away from the curb, overtaking, and parking among others in the on-road drive. The standard route we have designed does not systematically test for these skills and previous research has shown them to occur too infrequently for meaningful analysis. However, we included overall errors in our analyses which included these rare events as well as the more frequently occurring categories listed above. Note that some of the measures were only available in one platform.

## 3. Results and Discussion

### 3.1. Age-Group Differences across the Two Platforms

Table 2 shows the descriptive statistics for the composite measures for older and younger drivers from the simulated drives and the corresponding measures collected during the on-road drive. We conducted one-way between-subject ANOVA on the driving task composite measures obtained from each platform. Homogeneity of variance assumption was not violated for any of the simulator measures (max F(1,77) = 3.16, min *p* = 0.079). This assumption was violated for the on-road drive for lane keeping/observance errors F(1,60) = 11.01, *p* = 0.002) but not other measures (max F(1,59) = 3.11, min *p* = 0.083). The corresponding nonparametric Mann-Whitney U test indicated that the null hypothesis of no difference should be rejected in all cases where homogeneity of variance assumption was violated.

There were significant age differences in lane change, lane keeping, speed control, and overall performance in both the simulator and the on-road drive. Older drivers performed more poorly compared to younger drivers. There were no age differences in stopping or turning in either platform. Those findings are consistent with strong relative validity in that the pattern of significant and non-significant differences replicated across platforms with a rejection rate of 57%, 8 out of 14 tests were significant at alpha = 0.05, four in each platform and the same four tasks in each platform. This is even more impressive in that no special effort was made to ensure the item content of performance metrics for each driving task aligned with the safety error classifications used by the certified driving instructor in the on-road drive.

### 3.2. Similarity in Relative Rank-Order across the Two Platforms

Table 3 shows the Pearson correlations and associated significance levels. One outlier case sometimes changed the significance level of the correlations. In those instances, the magnitude of the correlation is provided both with the case included and with the case excluded, in parentheses. There are five task-specific measures that have similar interpretations across the two platforms, while Incursions (in the simulator) and Traffic Sign errors (on the road) are only measured in one of the platforms. Hence, when looking at the cross-platform correlations in Table 3, the diagonal elements corresponding to those five measures, plus the correlation between total number of errors (on-road) and overall performance (in the simulator) are of particular interest from a validity standpoint and have been highlighted in bold. These bolded correlations in the diagonal of Table 3 highlight the degree of convergence for comparable measures of performance across the two platforms. Examination of those bolded cells show that lane change and lane keeping but not speed control, stopping, or turning showed moderate correlations across the two platforms. These correlations suggest that specificity in convergence in relative rank-order on a task-by-task basis (the bolded diagonal elements of Table 3) was limited to two tasks. However, overall performance measure from both platforms showed a moderate correlation. Hence, overall rejection rate for the six diagonal elements was 50% (three out of six bolded diagonal elements are significant). In addition, 13 out of 31 off-diagonal elements of Table 3 among task-specific performance measures showed fair to moderate correlations with a *p*-value of 0.10 or better. Significant correlations in the off-diagonal elements are above chance (42% rejection rate) and indicate performance measures in the simulator predict on-road safety, but not on a by-task basis. For example, lane change performance in the simulated drives moderately predicted lane keeping and speed control in the on-road drive. Reaction time measures during incursion scenarios moderately predicted safety errors associated with turns in the on-road drive. Finally, overall performance score in the simulator predicted all specific error categories from the on-road drive with the exception of stopping errors.

Collectively, the pattern of correlations was consistent with a moderate degree of relative validity. While the overall performance metric indicated moderate convergence in relative rank-order between the two platforms, only two specific metrics of performance, lane change and lane keeping, correlated with the corresponding measure from the other platform. These two measures also correlated with other task performance measures. For example, lane keeping in the simulator was associated with traffic sign errors in the on-road drive as well as speed control and lane change. Measures that were only applicable to one platform such as reaction time in incursions and traffic sign errors in the on-road drive correlated with the overall performance metric in the other platform. Hence, unlike the age-group differences already presented, the pattern of correlations did not suggest a consistent pattern of specificity in the associations on a per task basis.

## 4. Conclusions

We examined the relative validity of performance in specific driving tasks measured in a fixed-base, immersive, full cabin DriveSafety simulator compared to safety errors committed during a 45-min standard on-road drive in an instrumented vehicle. The specific metrics of performance included lane changing, lane keeping, speed control, stopping, turning, and reaction time during incursions in addition to overall safety. The sample included healthy aging older drivers from the community with an average of 77 years and younger drivers, also from the community with an average age of 35 years. The range of performances on the screening instrument, MoCA, included those with mild cognitive impairment but not dementia indicating a range of functioning in the sample.

Older drivers performed more poorly than younger drivers on lane change, lane observance, speed control, and overall safety in both the simulator and the on-road drive. Furthermore, performance differences between the two age groups on stopping and turning tasks failed to be significant in both platforms. Hence, the pattern of mean differences that differentiated at-risk older drivers from the comparison group replicated both in terms of significant and non-significant differences across the two platforms. Correlational analyses indicated a moderate degree of alignment in rank-order of specific and overall performance metrics across the two platforms. However, compared to mean-difference analyses these analyses showed far lower levels of specificity on a task-by-task basis. For example, while performance measures in lane change, lane keeping, and overall safety correlated across the two platforms, these measures also correlated with performance in other tasks. Similarly, speed control did not correlate across the two platforms but it was correlated with lane change errors in the on-road drive. Together, those findings showed that performance in specific driving tasks including speed control and turning have moderate relative validity while lane change, lane keeping, and overall performance metrics have strong relative validity. To our knowledge this is the first study to demonstrate that driving safety assessed in the real-world with a standard on-road drive aligns moderately with simulator based assessments of safety in terms of specific driving tasks as well as overall safety.

Larger sample sizes would have permitted stronger tests for varying degrees of support for relative validity in each task. For example, larger sample sizes would permit an examination of correlations separately in the at-risk group of older drivers and the comparison group of younger drivers. Similarly, larger samples sizes would have permitted formal pattern tests on whether the magnitude of convergence is similar for specific and overall safety metrics. Nevertheless, the findings encourage future research to examine correspondence between simulator and real-world safety in more specific terms and suggest that a full-cabin fixed based DriveSafety simulator can be a useful assessment tool in evaluating the overall safety and some specific aspects of driving safety in aging populations. Our findings do not inform whether the moderate to strong relative validity we observed in this data set would transfer to the real world in a training or rehabilitation setting [20]. It also remains to be seen whether the relative validity we observed would generalize to other at-risk populations such as newly licensed teens. If in fact future research supports the moderate relative validity we observed in this study across the lifespan, driving simulators can fulfill an important promise in assessing driving safety without expensive on-road assessments and may help prevent motor vehicle crashes.

## Figures and Tables

**Table 1:** Description of performance metrics from the simulator and the on-road drive in each driving task.

Driving Tasks	Simulator-Based Measures		On-Road Drive
	Evaluation of Video for Safety in each Scenario	Vehicle Data	
Lane change (lane change on straightaways, merging on/off ramp)	Number of rejected gaps, failure to use side-view and rear-view mirrors, number of hesitations (steering wheel reversals)	Gap size, time to complete lane change, type of gap (2-sided *vs.* other)	Failure to signal correctly, improper speed control during various stages of the task (on/off ramp; interstate/highway); blind spot monitoring
Lane keeping (straight and curved)	N/A	Standard deviation of lane position, total time off-center by more than 0.51 meters, time to correct off-center drifts	Touching/straddling, lane line, center line, hits curb
Speed control	N/A	Time to match posted speed limit within +/− 5 mph	Difference in speed from posted limit by more than +/− 5 mph
Stopping (4-way, 2-way, or light-controlled intersection)	Incorrect location for the stop, rolling stops, running red lights	N/A	Incorrect location for stops, rolling stops, failure to yield the right of way, abrupt stops
Turning (4-way, 2-way, or light-controlled intersections)	Failing to scan the intersection, failure to follow the rules of right of way, incorrect signaling, erratic turn arc	N/A	Wide/sharp turns, failure to signal correctly, failure to yield the right of way
Incursions (side and front)	Collisions, reaction time to incursions (brake/steer)	N/A	N/A
Traffic Sign Compliance	N/A	N/A	Failure to complete stop during right turns on red lights, entering an intersection on red light, stopping in the intersection

**Table 2:** Descriptive statistics for older and younger groups for composite measures of driving performance in the simulator and on-road instrumented vehicle platforms.

Driving Task Composites	Simulated Drive ^1^			On-Road Drive ^2^		
	Younger	Older	*p*-Value	Younger	Older	*p*-Value
Lane Change	−0.10 (0.31)	0.14(0.27)	0.001	1.88 (1.85)	4.07 (1.93)	0.001
Lane Keeping ^3^	−0.16 (0.65)	0.33 (0.82)	0.004	4.44 (2.58)	7.03 (4.97)	0.001
Speed Control	−0.29 (0.67)	0.38 (1.04)	0.001	1.97 (1.99)	3.83 (2.55)	0.001
Stopping	−0.04 (0.35)	0.07 (0.38)	0.176	3.28 (2.10)	2.93 (1.58)	0.557
Turning	0.03 (0.63)	0.11 (0.40)	0.482	4.41 (2.05)	4.79 (2.32)	0.381
Traffic sign errors	n/a	n/a		1.59 (1.34)	0.187 (1.83)	0.504
Incursions	−0.07 (0.46)	0.15 (0.60)	0.089	n/a	n/a	
Overall performance	−0.11 (0.25)	0.21 (0.04)	0.001	18.34 (6.88)	25.48 (9.50)	0.001

^1^ N = 42 *vs.* 37 in simulator; ^2^ N = 32 *vs.* 30 in the instrumented vehicle; ^3^ the *p*-value is from the Mann-Whitney U test for the on-road test measures of lane keeping/observance.

**Table 3:** Pearson correlations of specific and overall performance metrics across simulator and on-road drive platforms.

Simulator Drive Measures	On-Road Drive Safety Errors ^1^						
	Lane Change Errors	Lane Observance Errors	Speed Control Errors	Stopping Errors	Turning Errors	Traffic Sign Errors	Total Safety Errors
Lane Change	0.36 **	0.35 **	0.40 **	−0.10	0.23 +	0.18	0.42 ***
Lane Keeping	0.24 + (0.26 *)	0.37 ** (0.46 ***)	0.20 (0.26 *)	−0.17	0.11	0.25 *	0.31 * (0.40 **)
Speed Control	0.27 *	0.11	**0.16**	−0.12	−0.08	0.13	0.14
Stopping	0.01	0.26 * (0.24 +)	0.12	**0.13**	0.11	0.10	0.23 +(0.21)
Turning	0.10	0.09	0.18	−0.02	**0.22 +**	0.12	0.19(0.23 +)
Incursions	0.30 *	0.22 + (0.28 *)	0.18	−0.10	0.33 **	0.20 (0.21 +)	0.33 ** (0.38 ***)
Overall performance	0.38 **	0.39 ** (0.47 ***)	0.34 **	−0.15	0.21 + (0.26 *)	0.29 * (0.32 *)	0.44 *** (0.52 ***)

^1^ N = 62 or (61) when one outlier is removed; + *p* < 0.10, * *p* < 0.05, ** *p* < 0.01, *** *p* < 0.005; n/a = not applicable.
